# Mitochondrial convergence of ferroptosis and cuproptosis in castration-resistant prostate cancer: From metabolic vulnerabilities to therapeutic targeting

**DOI:** 10.1016/j.bbadva.2026.100199

**Published:** 2026-08-03

**Authors:** Can-lin Wang, Jin-ting Lv, Si-jia Liu, Yang-yang Zhang, Shu-li Wang, Shu-qi Song

**Affiliations:** a Guang'anmen Hospital, China Academy of Chinese Medical Sciences, Beijing, 100053, China; b Tianjin Medical University, Tianjin, 300203, China; c Xiyuan Hospital, China Academy of Chinese Medical Sciences, Beijing, 100091, China

**Keywords:** Mitochondria, Ferroptosis, Cuproptosis, Castration-resistant prostate cancer, Therapeutic target

## Abstract

Castration-resistant prostate cancer (CRPC) represents the lethal stage of prostate cancer progression and is characterized by persistent therapeutic adaptation, androgen receptor (AR) signaling rewiring, and profound metabolic reprogramming. In recent years, ferroptosis and cuproptosis, two forms of metal-dependent regulated cell death, have emerged as potentially important therapeutic vulnerabilities in CRPC. Ferroptosis is primarily driven by iron-dependent lipid peroxidation, whereas cuproptosis is characterized by copper-induced proteotoxic stress of mitochondrial lipoylated proteins and consequent metabolic collapse. Although these two cell death modalities are mechanistically distinct, accumulating evidence suggests that mitochondria may serve as a critical point of convergence linking them. Mitochondrial metabolic dependency, iron–sulfur cluster homeostasis, lipoylation pathways, membrane organization, and mitochondrial quality control networks collectively shape the susceptibility of CRPC cells to ferroptosis and cuproptosis. In this review, we summarize the mitochondrial basis of ferroptosis and cuproptosis in CRPC and propose that their relationship is better understood as a shared mitochondrial vulnerability architecture rather than as an identical terminal execution pathway. Furthermore, we discuss how mitochondrial metal-death vulnerability may represent a therapeutically exploitable architecture in CRPC, providing a rationale for combination strategies that target ferroptosis, cuproptosis, and mitochondrial metabolic dependency.

## Highlights

• CRPC develops strong mitochondrial dependency under therapeutic pressure.
• Ferroptosis and cuproptosis are distinct but mitochondria-centered death programs.
• Fe–S homeostasis, Lipid peroxidation, lipoylation, and MQC shape metal-death sensitivity.
• Mitochondrial vulnerability defines a therapeutic window for metal-death targeting.

Prostate cancer is the second most commonly diagnosed malignancy in men worldwide [1], and its global disease burden is expected to rise steadily, with the number of new cases projected to reach approximately 2.9 million by 2040 [2]. Although androgen deprivation therapy (ADT) and androgen receptor signaling inhibition strategies represented by enzalutamide, abiraterone, and apalutamide have significantly prolonged patient survival, most patients with advanced disease eventually progress to CRPC within 18–24 months after treatment, a stage that is typically associated with markedly worsened clinical outcomes. It has been reported that the median overall survival thereafter is approximately 36 months [3,4]. This stage reflects the cumulative consequence of long-term selective pressure, under which tumor cells acquire resistance through multiple mechanisms, including reactivation of AR signaling, AR amplification and mutation, emergence of AR splice variants, compensatory bypass signaling, and lineage plasticity [5]. A prominent feature of CRPC is the enhancement of mitochondrial metabolic plasticity, which becomes progressively reinforced during disease evolution. This is manifested by coordinated reprogramming of glycolysis, lipid metabolism, one-carbon metabolism, and mitochondrial oxidative metabolism, thereby supporting tumor cell survival, malignant progression, and therapeutic adaptation [6,7]. This mitochondria-centered metabolic remodeling not only sustains the adaptive survival of CRPC but also provides a new mechanistic context for metal-dependent cell death.

**Fig. 1:**
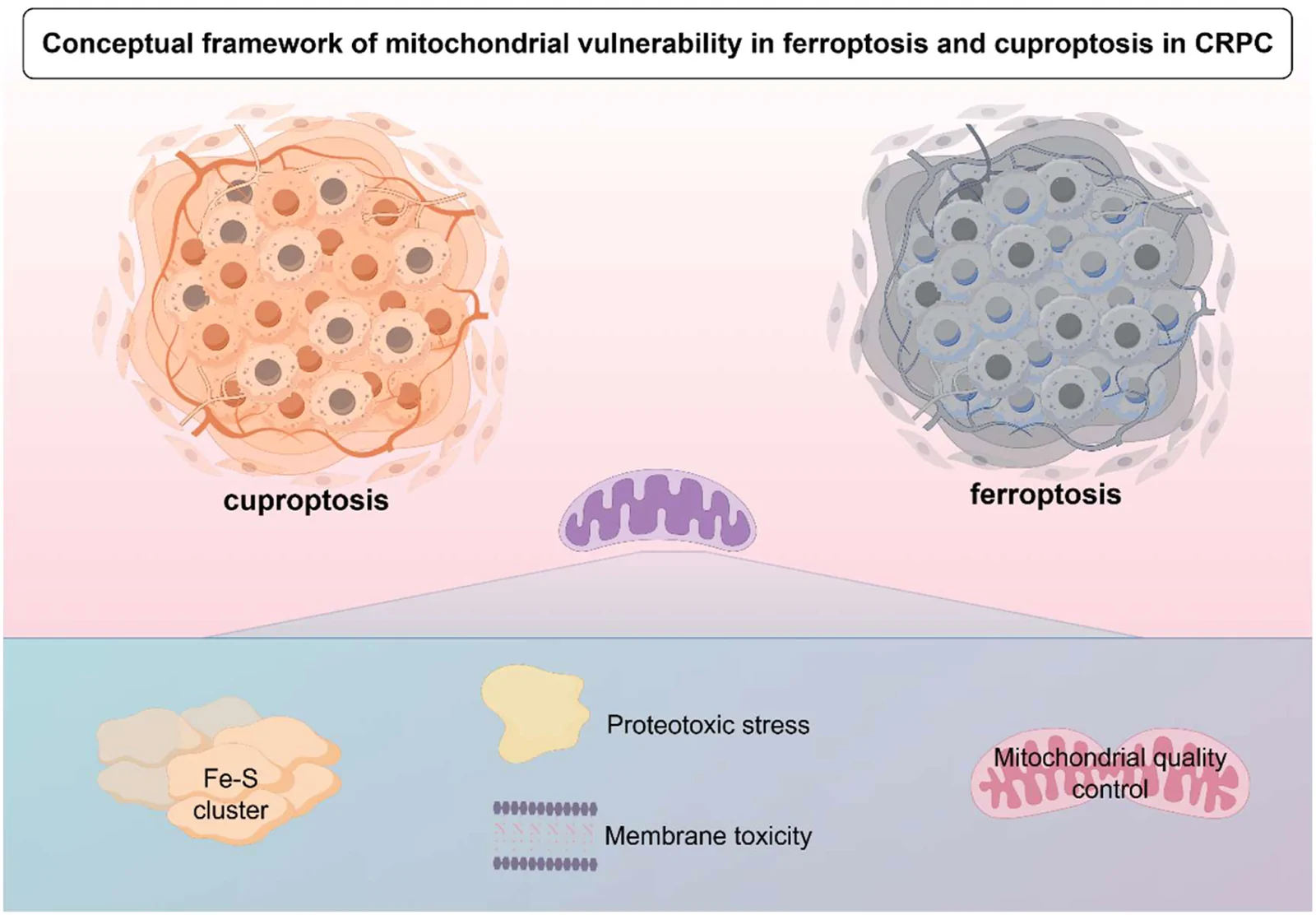
Conceptual framework of mitochondrial vulnerability in ferroptosis and cuproptosis in CRPC. Mitochondria may provide a candidate convergence interface linking ferroptosis and cuproptosis in CRPC through Fe–S cluster homeostasis, protein lipoylation, lipid peroxidation, and mitochondrial quality control. By Figdraw.

Although ferroptosis and cuproptosis are mechanistically distinct terminal death programs, they may share a mitochondria-centered vulnerability background in CRPC. This relationship is better understood as mitochondrial convergence based on common metabolic and homeostatic dependencies. Ferroptosis is centered on the uncontrolled propagation of iron-dependent lipid peroxidation, whereas cuproptosis is characterized by copper-induced aggregation of mitochondrial lipoylated proteins, destabilization of iron–sulfur cluster proteins, and subsequent metabolic collapse, with both processes being under substantial mitochondrial control [8,9]. This convergence may be particularly relevant in CRPC, where inhibition of AR signaling is accompanied by a further increase in mitochondrial dependency. In this setting, mitochondria may function not only as a key mechanistic hub linking metal-homeostasis imbalance to therapeutic vulnerability, but also as a central determinant of tumor-cell susceptibility to both ferroptosis and cuproptosis. Their relationship is therefore better understood as a shared mitochondrial vulnerability architecture, jointly constrained by mitochondria-related metabolic dependency, iron–sulfur cluster homeostasis, lipoylation pathways, membrane organization, and mitochondrial quality control networks. From this perspective, the present review focuses on the intrinsic connections among mitochondria, ferroptosis, and cuproptosis in CRPC, with particular emphasis on their potential points of mechanistic convergence. This framework may not only refine our understanding of therapeutic adaptation in CRPC, but also provide a conceptual basis for identifying new opportunities for combination treatment. Through this review, we aim to integrate these two forms of “metal-dependent cell death” from previously fragmented mechanistic concepts into a unified analytical framework capable of explaining metabolic reprogramming, resistance progression, and therapeutic vulnerability in CRPC (Fig. 1).

## 1 Mitochondrial reprogramming in CRPC creates a permissive background for metal-dependent cell death

Throughout the different stages of tumor growth and progression, mitochondria in cancer cells undergo complex functional and structural dynamic alterations, and cancer-cell survival is broadly dependent on the maintenance of mitochondrial integrity and function [10]. Through oxidative phosphorylation coupled to the tricarboxylic acid (TCA) cycle, mitochondria continuously generate ATP to meet the energetic demands required for cellular growth [11]. At the same time, multiple intermediates derived from the TCA cycle can be diverted into the biosynthetic pathways of lipids, amino acids, and nucleotides [12]. In certain tumor contexts, mitochondrial fatty acid β-oxidation is further enhanced, generating acetyl-CoA, NADH, and FADH2 to support TCA cycle activity, oxidative metabolism, and stress adaptation [13,14]. Meanwhile, mitochondrial glutamine catabolism is frequently reprogrammed: on the one hand, it provides carbon and nitrogen sources for the synthesis of amino acids and nucleotides; on the other hand, it replenishes the TCA cycle through glutamate/α-ketoglutarate anaplerosis [15]. Collectively, these metabolic networks sustain persistent tumor proliferation and survival, and in specific contexts, further promote disease progression and metastasis [16].

### 1.1 Fundamental features of mitochondrial metabolic remodeling in CRPC

Prostate epithelial cells undergo a distinctive metabolic reprogramming during the early stages of carcinogenesis (Table 1). Compared with adjacent normal tissues, prostate cancer tissues exhibit increased levels of TCA cycle enzymes and intermediate metabolites [[17], [18], [19]]. Intracellular zinc levels are markedly reduced in prostate cancer cells, resulting in the reactivation of mitochondrial aconitase, citrate oxidation, the TCA cycle, and oxidative phosphorylation (OXPHOS) [20]. A high mitochondrial content in prostate cancer specimens has been positively associated with disease progression and serves as an effective predictor of poor clinical outcome [21]. In prostate cancer cells that acquire resistance to standard therapies, such as enzalutamide and docetaxel, a metabolic shift from glycolysis toward OXPHOS can be observed [22].As disease progression continues under persistent therapeutic pressure, this mitochondrial involvement further evolves into a state of heightened metabolic plasticity, such that CRPC can no longer be viewed merely as a resistant stage defined by AR reactivation, but rather as an adaptive state sustained by coordinated support from mitochondrial metabolism, redox networks, and homeostatic maintenance programs [[23], [24], [25], [26]]. The metabolic phenotype of CRPC is typically characterized by profound remodeling in several major aspects. First, OXPHOS is upregulated: CRPC cells increasingly rely on OXPHOS as a major energy source through enhanced expression of mitochondrial electron transport chain (ETC) complexes, increased mitochondrial DNA (mtDNA) copy number, and/or augmented flux of metabolic substrates, such as glutamine and fatty acids, into the TCA cycle [27,28]. Second, mitochondrial biogenesis is reinforced: the expression of transcriptional coactivator PGC-1α and transcription factors NRF1/2 is elevated, thereby promoting mitochondrial biogenesis and network fusion [29]. Third, metabolic flexibility is increased: CRPC cells efficiently utilize a wide range of substrates, including lactate, ketone bodies, and fatty acids, to sustain continuous TCA cycle activity and OXPHOS [30,31]. Fourth, antioxidant capacity is extensively remodeled: to counterbalance the rise in reactive oxygen species (ROS) associated with enhanced OXPHOS, CRPC cells upregulate multiple mitochondrial antioxidant systems, including SOD2, PRX3, TRX2, and the GSH/GPx system, thereby establishing a new steady state characterized by high metabolic activity coupled with high antioxidant capacity [[32], [33], [34], [35]]. Collectively, these features define the fundamental landscape of mitochondrial metabolic remodeling in CRPC and confer an unprecedented dependence on mitochondrial function.

**Table 1:** Mitochondrial metabolic remodeling during prostate cancer progression and therapy resistance.

**Stage**	**Metabolic feature**	**Functional consequences**	**Ref.**
**Early** **carcinogenesis**	**TCA-cycle enzymes and intermediates ↑; Intracellular zinc levels ↓**	Citrate oxidation ↑ TCA-cycle activity ↑ OXPHOS ↑	[[17], [18], [19], [20]]
**Therapy** **resistance**	**Coordinated interaction of mitochondrial metabolism, redox networks, and homeostatic maintenance programs**	An adaptive state	[[23], [24], [25], [26]]
**CRPC**	**ETC-complex expression ↑** **mtDNA copy number ↑** **Substrate flux ↑**	OXPHOS↑	[[27], [28]]
	**PGC-1α expression ↑** **NRF1/2 expression ↑** **Enhanced network fusion**	Mitochondrial biogenesis↑	[29]
	**Utilization of lactate, ketone bodies, and fatty acids sustains TCA-cycle and OXPHOS,**	Metabolic flexibility↑	[30,31]
	**SOD2, PRX3, TRX2 ↑** **GSH/GPx system ↑**	Antioxidant capacity↑	[[32], [33], [34], [35]]

### 1.2 AR signaling, therapeutic pressure, and the reinforcement of mitochondrial dependency

In prostate cancer, the AR programs global metabolic pathways, including aerobic glycolysis, mitochondrial respiration, and de novo lipogenesis, thereby supporting the metabolic and biosynthetic demands of tumor cells [36,37]. AR signaling is not only the central driver of prostate cancer growth, but also a key determinant of its metabolic architecture. AR regulates glucose, lipid, amino acid, and nucleotide metabolism, and continuously shapes the energetic landscape of prostate cancer by influencing citrate fate, fatty acid synthesis, and mitochondrial substrate supply [38,39]. With the implementation of castration-based therapies and AR pathway inhibitors, tumor cells lose part of the canonical AR transcriptional program while being simultaneously forced to reconstruct survival strategies under newly imposed metabolic constraints. Therapeutic pressure therefore substantially contributes to the emergence of a renewed state of mitochondrial dependency. Studies have shown that AR inhibition reduces glycolytic activity, induces marked mitochondrial elongation, and increases reliance on mitochondrial oxidative metabolism. At the same time, AR-blocked cells become more sensitive to complex I inhibition, indicating that treatment itself can create a mitochondria-centered metabolic vulnerability. This work further identified DRP1-mediated mitochondrial dynamics and MYC signaling as important regulators of this state transition [40]. Consistent with this view, enzalutamide has been shown to increase tumor-cell dependence on mitochondrial metabolism and, in parallel, to markedly enhance susceptibility to copper-mediated cell death, suggesting that mitochondrial remodeling following AR inhibition is not merely an accompanying event in the development of resistance, but may also represent a prerequisite for metal-dependent cell death [41].

### 1.3 Mitochondria as an integrative hub for metabolic, redox, and metal homeostasis

In the context of mitochondrial reprogramming, mitochondria constitute a critical entry point for understanding metal-dependent cell death in CRPC, not merely because they are responsible for ATP production, but because they simultaneously integrate metabolic homeostasis, redox homeostasis, and metal homeostasis [42,43] (Table 2). From a metabolic perspective, mitochondria host the TCA cycle, the electron transport chain (ETC), and multiple substrate oxidation processes, thereby serving as the central node that determines whether a cell can maintain a high-OXPHOS state [44,45]. From a redox perspective, mitochondria are not only a major source of reactive oxygen species (ROS), but also the principal intracellular arena in which buffering systems related to glutathione, NADPH, and coenzyme Q exert their protective effects [42,46]. From the perspective of metal homeostasis, iron is indispensable for mitochondrial respiration, heme synthesis, and iron–sulfur (Fe–S) cluster biogenesis [47,48], whereas copper is closely linked to the function of the mitochondrial respiratory chain and, when present in excess, can trigger cuproptosis [49]. In other words, mitochondria are intrinsically equipped with the structural and functional basis to couple substrate utilization, oxidative stress, and metal handling. This integrative property is particularly important in CRPC. CRPC is not simply characterized by enhanced metabolism; rather, under persistent therapeutic pressure, it maintains a state of “high stress yet sustained viability.” In this state, tumor cells require sufficient mitochondrial activity to support growth and therapeutic resistance, while simultaneously avoiding excessive ROS accumulation, metal disequilibrium, and membrane damage that would otherwise push them beyond the lethal threshold. It is precisely within this fragile balance that mitochondria determine whether metal perturbations are buffered and absorbed, or instead amplified and translated into lethal outputs such as ferroptosis or cuproptosis.

**Table 2:** Comparison of mitochondrial links between ferroptosis and cuproptosis in CRPC.

**Dimension**	**Shared mitochondrial basis**	Ref.
Metabolic platform	Mitochondria sustain the TCA cycle, ETC,OXPHOS, ATP production, and substrate oxidation.	[[44], [45]]
Redox buffering	Mitochondrial ROS is restrained by GSH-, NADPH-, and CoQ-linked antioxidant systems.	[42,46]
Metal handling	Fe supports mitochondrial respiration, heme synthesis, and iron–sulfur (Fe–S) cluster biogenesis. Cu supports respiratory-chain function, but excess Cu triggers cuproptosis.	[[47], [48], [49]]

### 1.4 Experimental criteria for defining ferroptosis and cuproptosis

Because ferroptosis and cuproptosis may both be accompanied by ROS accumulation, mitochondrial dysfunction, and reduced cell viability, mechanism-specific criteria are required to distinguish these regulated cell death modalities from nonspecific oxidative or mitochondrial injury [9,50]. Ferroptosis should be defined as an iron-dependent, lipid peroxidation-driven form of regulated cell death rather than a simple increase in oxidative stress [51]. Its identification should therefore integrate three levels of evidence: molecular markers, biochemical or functional assays, and pharmacological rescue experiments [52].At the molecular level, ferroptosis is commonly associated with impairment of the SLC7A11/system Xc⁻–GSH–GPX4 antioxidant axis [53]. Reduced SLC7A11 expression or activity indicates impaired cystine uptake and decreased GSH synthesis, whereas GPX4 downregulation or inactivation reflects defective detoxification of phospholipid hydroperoxides. ACSL4 is another important marker because it enriches cellular membranes with PUFA-containing phospholipids, thereby increasing the availability of oxidizable lipid substrates for ferroptosis [54]. At the biochemical level, lipid peroxidation should be directly evaluated using assays such as C11-BODIPY 581/591 oxidation, 4-HNE or MDA detection, and, when possible, oxidized phospholipid profiling by lipidomics [55]. C11-BODIPY 581/591 oxidation, detected by fluorescence microscopy or flow cytometry, is widely used to monitor lipid ROS accumulation during ferroptosis. Increased 4-HNE and MDA levels provide complementary evidence of lipid peroxidation products, whereas targeted lipidomics can further identify oxidized phospholipid species involved in ferroptotic execution. Iron dependency should be assessed by measuring labile iron or Fe²⁺ accumulation, together with changes in iron-handling proteins when appropriate. Importantly, ferroptotic death should be suppressible by ferroptosis inhibitors such as ferrostatin-1 or liproxstatin-1, or by iron chelators such as deferoxamine, which helps distinguish ferroptosis from apoptosis, necroptosis, general ROS injury, or nonspecific mitochondrial toxicity [56].

Cuproptosis should likewise be defined by its characteristic mitochondrial proteotoxic mechanism rather than by copper-induced cytotoxicity alone. Its key molecular features include FDX1-dependent copper reduction, LIAS-dependent mitochondrial protein lipoylation, copper binding to lipoylated TCA-cycle proteins, aggregation of lipoylated DLAT/DLST-containing protein complexes, loss or destabilization of Fe–S cluster proteins, and induction of proteotoxic stress. These events can be examined by measuring FDX1 and LIAS expression or activity, detecting lipoylated DLAT/DLST by immunoblotting, assessing high-molecular-weight DLAT/DLST-containing aggregates under non-reducing or semi-denaturing conditions, and monitoring Fe–S cluster protein stability. Because cuproptosis depends on an active mitochondrial metabolic state, functional assays of mitochondrial respiration, such as oxygen consumption rate, TCA-cycle activity, mitochondrial membrane potential, and sensitivity to respiratory chain inhibition, are also important. Copper dependency should be confirmed by rescue with copper chelators, such as tetrathiomolybdate or bathocuproine disulfonate, and ideally by genetic or pharmacological modulation of FDX1, LIAS-mediated lipoylation, or mitochondrial respiration [9]. Therefore, in CRPC studies, claims of ferroptosis or cuproptosis should be supported by convergent evidence from molecular markers, functional assays, and rescue experiments [57]. This evidence-based classification is essential to avoid misclassifying nonspecific oxidative stress, mitochondrial dysfunction, or general cytotoxicity as true metal-dependent regulated cell death.

## 2 Distinct mitochondrial bases of ferroptosis and cuproptosis in CRPC

### 2.1 Mitochondrial basis of ferroptosis in CRPC

**Fig. 2:**
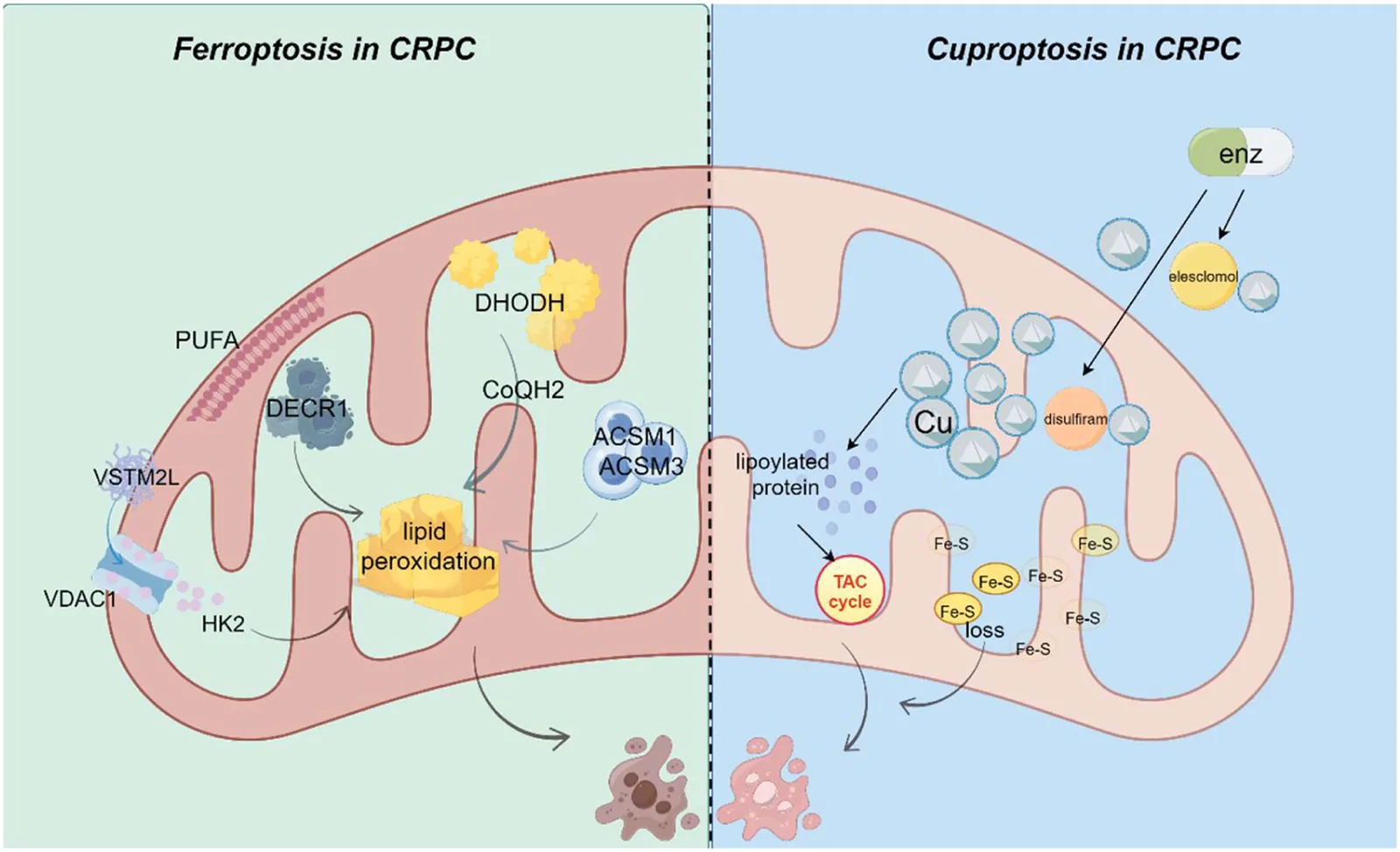
Fig. 2**a** Mitochondrial anti-ferroptotic defense is coordinated by several protective modules. DHODH suppresses mitochondrial lipid peroxidation through regeneration of reduced coenzyme Q. DECR1 promotes PUFA β-oxidation–dependent PUFA catabolism, and ACSM1/3 sustain fatty acid oxidation and energy production, thereby limiting mitochondrial oxidative stress and lipid peroxidation. Meanwhile, mitochondria-localized VSTM2L maintains mitochondrial homeostasis by strengthening HK2–VDAC1 interaction and preventing VDAC1 oligomerization, ultimately inhibiting ferroptosis in prostate cancer cells. Fig. 2**b** Copper overload induces cuproptosis by binding to lipoylated TCA cycle proteins, triggering their aggregation, loss of Fe–S cluster proteins, proteotoxic stress, and metabolic collapse. Enzalutamide heightens mitochondrial metabolic dependence in prostate cancer cells, thereby increasing susceptibility to copper ionophore–induced cell death, including that triggered by elesclomol or disulfiram. By Figdraw.

At the mitochondrial level, ferroptosis in CRPC should not be viewed merely as a passive consequence of lipid peroxidation, but rather as a regulated death process shaped jointly by mitochondrial antioxidant defenses, fatty acid oxidation programs, and membrane structural homeostasis (Fig. 2). First, dihydroorotate dehydrogenase (DHODH), located on the inner mitochondrial membrane, has been shown to suppress mitochondrial lipid peroxidation through regeneration of reduced coenzyme Q. When this defense is compromised, peroxidation of mitochondrial membrane lipids is markedly amplified, thereby lowering the cellular tolerance threshold for ferroptosis [58,59] (Fig. 2**a**). This is particularly relevant in CRPC, where therapy-exposed cells often become more dependent on mitochondrial oxidative metabolism and are therefore increasingly reliant on intramitochondrial redox-buffering systems for survival.

Second, mitochondria determine substrate availability for ferroptosis through polyunsaturated fatty acid (PUFA) handling and fatty acid oxidation. DECR1, a mitochondrial auxiliary enzyme involved in PUFA β-oxidation and linked to prostate cancer cell survival, protects tumor cells from ferroptosis by facilitating PUFA catabolism. Inhibition of DECR1 results in impaired PUFA metabolism, accumulation of lipid peroxidation, and enhanced ferroptotic cell death [60]. Consistent with this, ACSM1 and ACSM3, both important regulators of fatty acid metabolism in prostate cancer, can likewise restrain mitochondrial oxidative stress, lipid peroxidation, and ferroptosis by promoting fatty acid oxidation and energy production; conversely, their loss leads to elevated mitochondrial oxidative stress and triggers ferroptotic cell death [61]. These findings indicate that, in CRPC, mitochondria are not simply passive recipients of ferroptotic injury, but actively preconfigure the substrate ecology of ferroptosis by directing PUFA fate and controlling oxidative pressure.

Third, mitochondrial regulation of ferroptosis also extends to membrane organization and outer membrane gating. Mitochondria-localized VSTM2L has been shown to maintain mitochondrial homeostasis by enhancing HK2–VDAC1 interaction and suppressing VDAC1 oligomerization, thereby inhibiting ferroptosis in prostate cancer cells. By contrast, downregulation of VSTM2L leads to loss of mitochondrial membrane potential, ROS accumulation, and increased susceptibility to ferroptosis. This finding suggests that, in CRPC, mitochondria regulate ferroptosis not only through metabolism and redox defense, but also through coordinated control of outer membrane channel conformation, membrane potential, and membrane structural integrity, which together determine the accessibility of ferroptotic execution [62].

Taken together, the mitochondrial basis of ferroptosis in CRPC is better conceptualized as a threshold-control system jointly governed by the CoQ antioxidant network, fatty acid oxidation programs, and membrane structural homeostasis.

### 2.2 Mitochondrial basis of cuproptosis in CRPC

Compared with ferroptosis, cuproptosis has exhibited a much more explicit mitochondrial dependency since its initial description. In 2022, Tsvetkov and colleagues first demonstrated that copper-dependent cell death is neither a conventional form of oxidative injury nor a collateral branch of apoptosis, but rather a distinct mode of cell death built upon mitochondrial respiration (Fig. 2**b**). Excess copper can directly bind to lipoylated proteins within the TCA cycle, inducing their aggregation, accompanied by loss of Fe–S cluster proteins, ultimately triggering proteotoxic stress and metabolic collapse. Thus, the occurrence of cuproptosis does not depend simply on “copper overload” per se, but on whether cells reside in a mitochondrial metabolic state characterized by dependence on the TCA cycle, lipoylated protein homeostasis, and Fe–S cluster integrity [9].In CRPC, enzalutamide markedly increases cellular dependence on mitochondrial metabolism and thereby enhances susceptibility to copper-dependent cell death induced by the copper ionophores elesclomol or disulfiram; notably, this effect can be reversed by copper chelation. More importantly, this phenomenon has been validated not only in in vitro cell models, but also in resistant models and in vivo experiments. These findings indicate that, in CRPC, the critical prerequisite for cuproptosis is not copper exposure alone, but rather therapy-induced reinforcement of mitochondrial dependency [41].The mitochondrial basis of cuproptosis in CRPC is therefore clearly distinct from that of ferroptosis. In ferroptosis, mitochondria primarily shape the death threshold through antioxidant defenses, fatty acid oxidation, and membrane homeostasis. By contrast, in cuproptosis, mitochondria themselves constitute the principal site of damage, and the likelihood of death is jointly determined by respiratory dependency, the abundance of lipoylated TCA proteins, and the stability of Fe–S proteins. In other words, the mitochondrial basis of cuproptosis in CRPC is better conceptualized as a metabolic–proteotoxic control system organized around a respiration scaffold, lipoylated proteins, and Fe–S homeostasis.

## 3 Mitochondrial convergence mechanisms linking ferroptosis and cuproptosis in CRPC

Although ferroptosis and cuproptosis are fundamentally distinct in their terminal execution mechanisms—with the former centered on iron-catalyzed lipid peroxidation and the latter characterized by copper-induced mitochondrial proteotoxic stress—both are highly dependent on mitochondrial metabolic activity. This shared metabolic prerequisite constitutes a deeper background determinant of CRPC cell sensitivity to these two forms of metal-dependent cell death. Metabolic reprogramming is a key driver of therapeutic resistance in CRPC [8,9]. Under the sustained selective pressure of androgen deprivation therapy, CRPC cells undergo multifaceted metabolic adaptation, thereby creating a permissive context for the induction of both ferroptosis and cuproptosis. Ferroptosis depends on the iron-catalyzed propagation of lipid peroxidation, a process that requires both abundant membrane PUFA substrates and ROS, both of which are closely linked to mitochondrial metabolism [8]. Mitochondria are not only a major intracellular source of ROS, but also a central hub for PUFA metabolism and iron–sulfur cluster biogenesis. By contrast, cuproptosis relies even more directly on mitochondrial respiratory activity: following reduction by FDX1, copper primarily targets lipoylated enzymes of the TCA cycle, such as DLAT and DLST, triggering their aberrant aggregation and functional collapse, which in turn provokes proteotoxic stress and cell death [9]. Accordingly, effective induction of cuproptosis requires an active mitochondrial metabolic state as a prerequisite. Notably, CRPC cells under ARSI pressure often exhibit enhanced mitochondrial metabolic activity, and this adaptive metabolic upregulation may place them within a shared window of vulnerability to both ferroptosis and cuproptosis. On the one hand, increased mitochondrial respiration generates higher levels of ROS, thereby providing the oxidative drive required for ferroptosis. On the other hand, an active TCA cycle implies increased abundance and activity of lipoylated enzymes, thereby supplying abundant protein substrates for copper-mediated targeting. This overlap in metabolic dependency may underlie the potential for synergistic therapeutic targeting of ferroptosis and cuproptosis in CRPC.

Importantly, we do not define ferroptosis and cuproptosis as identical terminal death programs, nor do we assume that they necessarily engage in direct bidirectional mechanistic interaction. Instead, we distinguish several levels of relationship between these two forms of metal-dependent regulated cell death. First, ferroptosis and cuproptosis may coexist within the same disease context, particularly in therapy-adapted CRPC cells with enhanced mitochondrial metabolic dependency. Second, they may share common upstream regulators, including mitochondrial respiration, redox buffering, Fe–S cluster homeostasis, protein lipoylation, metal handling, and mitochondrial quality control. Third, they may converge on mitochondrial vulnerability nodes, where perturbation of respiration, Fe–S cluster stability, or proteostasis can influence the susceptibility of CRPC cells to either death modality. These relationships should be understood primarily as mitochondrial convergence rather than as direct proof that one death pathway necessarily drives the other. Direct CRPC-specific evidence supporting such bidirectional amplification remains limited. Therefore, mitochondrial convergence refers to shared mitochondrial dependency and overlapping vulnerability nodes, whereas direct mechanistic interaction is considered a conditional and testable hypothesis requiring further validation in CRPC-specific models.This distinction is important for interpreting the therapeutic implications of ferroptosis and cuproptosis in CRPC. Combined targeting of ferroptosis and cuproptosis should not be understood as simply activating two identical death pathways. Rather, it may exploit a shared mitochondrial vulnerability architecture in which ARSI-induced metabolic dependency, Fe–S/lipoylation imbalance, redox stress, and mitochondrial quality-control failure jointly narrow the survival margin of CRPC cells.

### 3.1 Fe–S cluster homeostasis: a candidate mitochondrial convergence interface between ferroptosis and cuproptosis

**Fig. 3:**
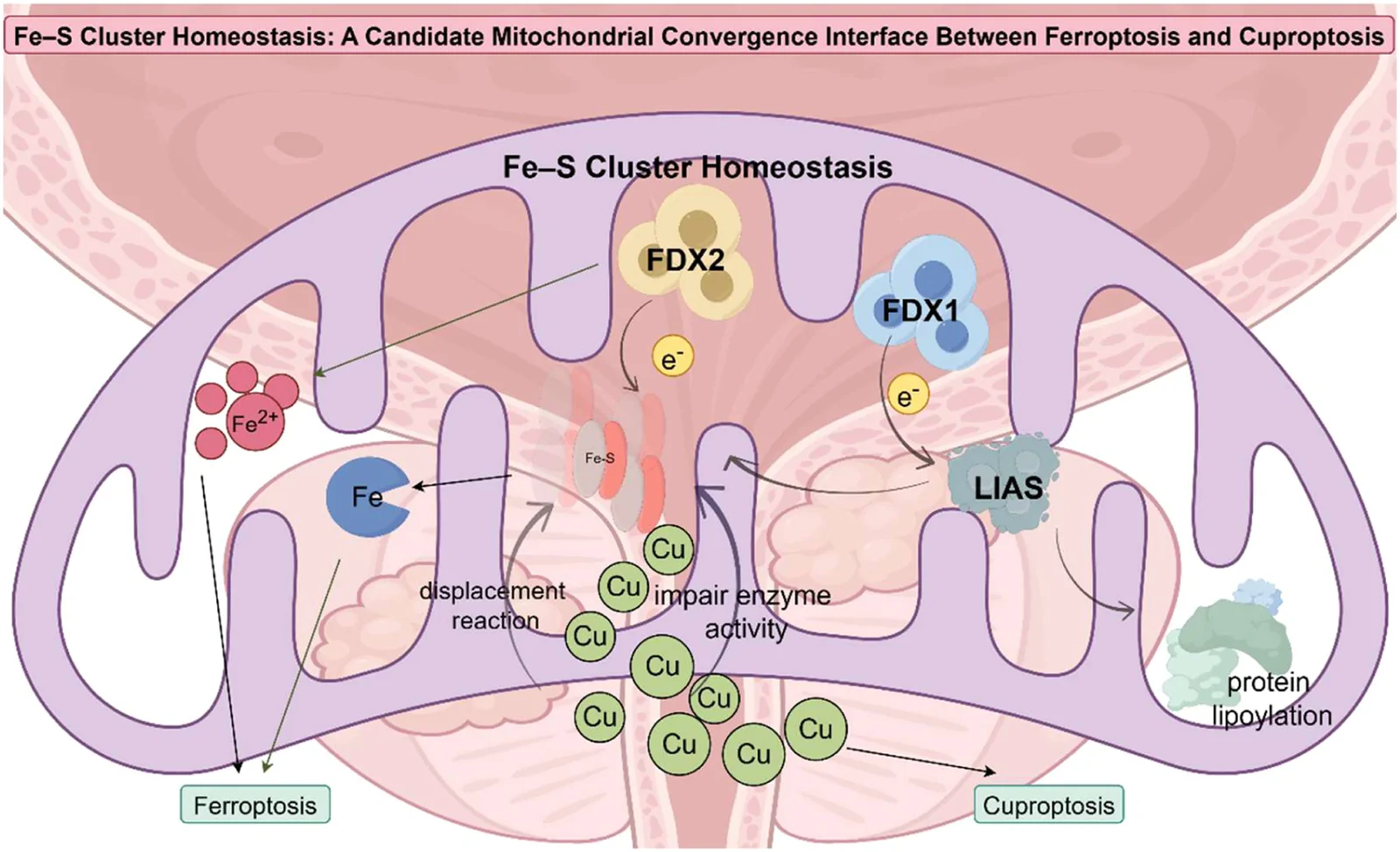
FDX2 supports Fe–S cluster biogenesis by providing electrons for Fe–S assembly, whereas the FDX1–LIAS axis drives mitochondrial protein lipoylation and couples lipoylation to Fe–S homeostasis. Copper overload disrupts Fe–S cluster integrity both by directly damaging Fe–S proteins and by suppressing Fe–S cluster biogenesis, leading to loss of Fe–S enzyme activity and mitochondrial dysfunction. Impaired Fe–S cluster maintenance may activate an iron-starvation response to promote ferroptosis. FDX2 deficiency may induce Fe²⁺ overload, and increase ferroptosis sensitivity in a context-dependent manner. By Figdraw.

Disruption of Fe–S cluster homeostasis represents a core molecular node linking ferroptosis and cuproptosis. The centrality of this intersection arises from the multiple roles of iron–sulfur clusters in mitochondrial metabolism and redox regulation. Ferredoxins FDX1 and FDX2 are representative members of the mitochondrial Fe–S protein family and play essential regulatory roles in maintaining mitochondrial redox homeostasis, electron transfer, energy metabolism, and Fe–S cluster biogenesis [[63], [64], [65]]. In human cells, FDX1 and FDX2 exhibit functionally distinct yet coordinated regulatory patterns: FDX1 is primarily involved in steroidogenesis, protein lipoylation, and copper redox cycling, whereas FDX2 serves as a core factor in Fe–S cluster assembly by providing the electrons required for Fe–S cluster biosynthesis [[63], [64], [65], [66]] (Fig. 3). LIAS (lipoic acid synthase) is itself an Fe–S protein, and its enzymatic activity depends on electrons supplied by FDX1. The FDX1–LIAS axis directly controls mitochondrial protein lipoylation and, through the dependence of LIAS on Fe–S cluster integrity, functionally couples protein lipoylation to Fe–S homeostasis [67]. The impact of copper overload on Fe–S cluster homeostasis is dual in nature. On the one hand, copper ions can directly react with Fe–S clusters, leading to destabilization and degradation of Fe–S proteins. On the other hand, copper overload can indirectly impair Fe–S enzyme activity by suppressing Fe–S cluster biogenesis, thereby causing mitochondrial dysfunction [9,68,69]. Furthermore, disruption of Fe–S cluster homeostasis may also influence ferroptosis sensitivity, although this connection has been validated mainly in non-CRPC tumor models. In lung cancer, suppression of the Fe–S cluster biosynthetic enzyme NFS1 limits Fe–S cofactor maintenance, activates an iron-starvation response, and cooperates with inhibition of cysteine transport or glutathione biosynthesis to trigger ferroptosis [70].AND deficiency of FDX2, a mitochondrial Fe–S cluster assembly factor, was shown in ovarian cancer cells to induce Fe²⁺ overload and sensitize cells to ferroptosis in a context-dependent manner [71]. This functional “bifurcation” between the two forms of cell death, determined by the divergent roles of FDX family members, nevertheless converges on the shared metabolic node of Fe–S cluster homeostasis. In CRPC cells, any disturbance in Fe–S cluster homeostasis—whether caused by direct copper-induced Fe–S cluster damage or by aberrant Fe–S assembly secondary to iron metabolic imbalance—may simultaneously influence the sensitivity of both death pathways [[71], [72]]. However, direct evidence demonstrating that cuproptosis-associated Fe–S cluster disruption releases labile iron and thereby amplifies ferroptotic lipid peroxidation in CRPC remains limited. In CRPC, the Fe–S-to-ferroptosis axis should be interpreted as a mechanistically plausible hypothesis rather than a fully validated pathway. Future CRPC-specific studies should directly test this hypothesis by manipulating FDX1, FDX2, LIAS, NFS1, and other Fe–S assembly factors, and by assessing labile iron accumulation,GPX4/SLC7A11 status, lipoylated protein aggregation, Fe–S protein stability, mitochondrial respiration, and rescue by ferroptosis inhibitors, copper chelators, or iron chelators.

### 3.2 Lipoylation-dependent proteotoxic stress and lipid peroxidation-dependent membrane toxicity

Although ferroptosis and cuproptosis are both characterized by oxidative damage at the terminal stage of cell death, they give rise to two mechanistically distinct yet metabolically interconnected forms of stress: lipoylation-dependent proteotoxic stress and lipid peroxidation-dependent membrane toxicity. The molecular basis of cuproptosis lies in the direct interaction of copper with lipoylated enzymes within the mitochondrial TCA cycle [9]. Under conditions of copper overload, FDX1 reduces Cu²⁺ to Cu⁺, which subsequently binds to lipoylated enzymes and induces their aberrant oligomerization, leading to the formation of high-molecular-weight protein aggregates [9]. Physiological lipoylation of TCA cycle enzymes, such as pyruvate dehydrogenase (PDH) and α-ketoglutarate dehydrogenase (α-KDH), is essential for maintaining efficient mitochondrial energy metabolism. Copper-induced aggregation of these lipoylated proteins disrupts TCA cycle function, provokes proteotoxic stress, and ultimately triggers cell death [9]. Cuproptosis is therefore defined as a copper-dependent form of regulated cell death driven by the direct targeting of lipoylated mitochondrial proteins and the consequent induction of proteotoxic stress, and its execution is strictly dependent on mitochondrial respiratory activity.By contrast, the mechanistic cascade of ferroptosis is centered on membrane lipid peroxidation. Iron-catalyzed Fenton chemistry generates highly reactive hydroxyl radicals (·OH), which attack PUFA-containing phospholipids in cellular membranes and initiate a self-propagating lipid peroxidation chain reaction. Once this oxidative damage accumulates beyond a critical threshold, membrane integrity and selective permeability are compromised, ultimately leading to lytic cell death [8].

Thus, cuproptosis primarily causes metabolic collapse by targeting mitochondrial lipoylated TCA proteins and eliciting proteotoxic stress, whereas ferroptosis is driven by peroxidation of membrane phospholipids and can affect multiple subcellular membrane systems [9,73]. Mitochondrial membrane lipid peroxidation, for example, may disrupt respiratory chain stability through cardiolipin oxidation and impair PDH/TCA function. Conversely, copper overload and cuproptosis can damage mitochondrial lipoylated enzymes while increasing mitochondrial ROS, thereby generating a metabolic and oxidative environment that favors ferroptotic oxidation [74,75]. On this basis, ferroptosis and cuproptosis may exhibit therapeutically exploitable synergy in CRPC, although this possibility still requires direct experimental validation. Notably, in prostate cancer, the fatty acid synthase inhibitor G28UCM has been shown to disrupt the stability of mitochondrial fatty acid synthesis (mtFAS) and succinate dehydrogenase subunit B (SDHB). On the one hand, this inhibitor suppresses cuproptosis in prostate cancer cells by downregulating LIAS expression; on the other hand, it increases labile iron, induces pseudohypoxic signaling, iron overload, and lipid peroxidation, thereby enhancing cellular sensitivity to ferroptosis [76]. These findings not only reveal the metabolic interconnection between the two types of toxic stress, but also suggest that, in certain contexts, either cuproptosis or ferroptosis may become the dominant death program. Importantly, CRPC-specific evidence further indicates that PDHA1 functions as a critical modulator of cuproptosis and enzalutamide sensitivity in CRPC. Mechanistically, PDHA1 increases intracellular acetyl-CoA, enhances H3K27 acetylation, and upregulates SLC7A11, thereby promoting cystine uptake and GSH synthesis. Elevated GSH then chelates intracellular copper, suppresses cuproptosis, and reduces the efficacy of enzalutamide. Notably, this PDHA1–SLC7A11–GSH axis may also be relevant to ferroptosis, because SLC7A11-mediated cystine uptake supports GSH synthesis, and GSH is required for GPX4-dependent detoxification of lipid hydroperoxides. Therefore, PDHA1-driven enhancement of the SLC7A11/GSH system may simultaneously strengthen copper-buffering capacity and reinforce anti-ferroptotic redox defense. Conversely, targeting PDHA1 restores cuproptosis and sensitizes CRPC cells to enzalutamide treatment, while it may also lower the GSH-dependent threshold against ferroptotic lipid peroxidation. However, whether PDHA1 inhibition directly promotes ferroptosis in CRPC remains to be experimentally validated [77].

### 3.3 Mitochondrial quality control: restricting death amplification or promoting resistant survival

The mitochondrial quality control (MQC) system comprises the mitochondrial unfolded protein response (UPR^mt^), mitophagy, and mitochondrial dynamics (fission and fusion). When activated in a moderate and coordinated manner, these pathways can limit the excessive amplification of metal-dependent cell death; however, when dysregulated, they may instead promote therapy-resistant survival of tumor cells [46]. More specifically, in the context of prostate cancer, mitophagy may also intersect with metal-dependent cell death. Ginsenoside Rh2 (GRh2) has been shown to induce mitochondrial dysfunction through membrane depolarization, increased ROS, and disruption of the ATP/ADP balance, thereby activating mitophagy. By simultaneously inducing mitophagy-associated mitochondrial injury and ferroptosis, GRh2 exerts a synergistic antitumor effect in prostate cancer (PC) [78]. In this setting, massive accumulation of autophagosomes may no longer represent a signal of adaptive repair, but rather reflect a state of “excessive clearance” or “ineffective clearance.” Such aberrant mitophagy may irreversibly degrade the mitochondrial network and release pro-oxidant factors, including labile iron, thereby shifting from a cytoprotective mechanism to a death-promoting clearance program that accelerates cellular collapse and amplifies ferroptotic signaling.By contrast, direct primary evidence linking MQC to cuproptosis in CRPC remains limited, although MQC pathways are theoretically well positioned to influence both the threshold and the magnitude of cuproptotic responses. Existing studies have shown that PINK1/Parkin-dependent mitophagy can suppress cuproptosis, and that hypoxia-induced autophagy similarly attenuates FDX1-mediated cuproptotic injury. Conversely, under conditions of impaired autophagy accompanied by enhanced Drp1-mediated mitochondrial fission, cellular susceptibility to cuproptosis may increase [[79], [80], [81]]. However, most of these findings have thus far been derived from models outside prostate cancer, and the direct mechanisms operating in CRPC remain to be clarified.

## 4 Therapeutic implications: combined strategies targeting mitochondrial convergent mechanisms

The therapeutic exploitation of ferroptosis and cuproptosis in CRPC should be interpreted according to evidence strength and model specificity. Although several strategies suggest that mitochondrial metabolic dependency may create a shared vulnerability to metal-dependent cell death, their levels of validation differ substantially. Therefore, we classify these therapeutic implications into four categories: strategies supported by direct CRPC or CRPC-related evidence, strategies supported by general prostate cancer models, strategies supported by non-prostate cancer models, and emerging hypotheses that require CRPC-specific validation.

### 4.1 Combined induction of ferroptosis and cuproptosis: translational rationale and evidence limitations in CRPC

**Fig. 4:**
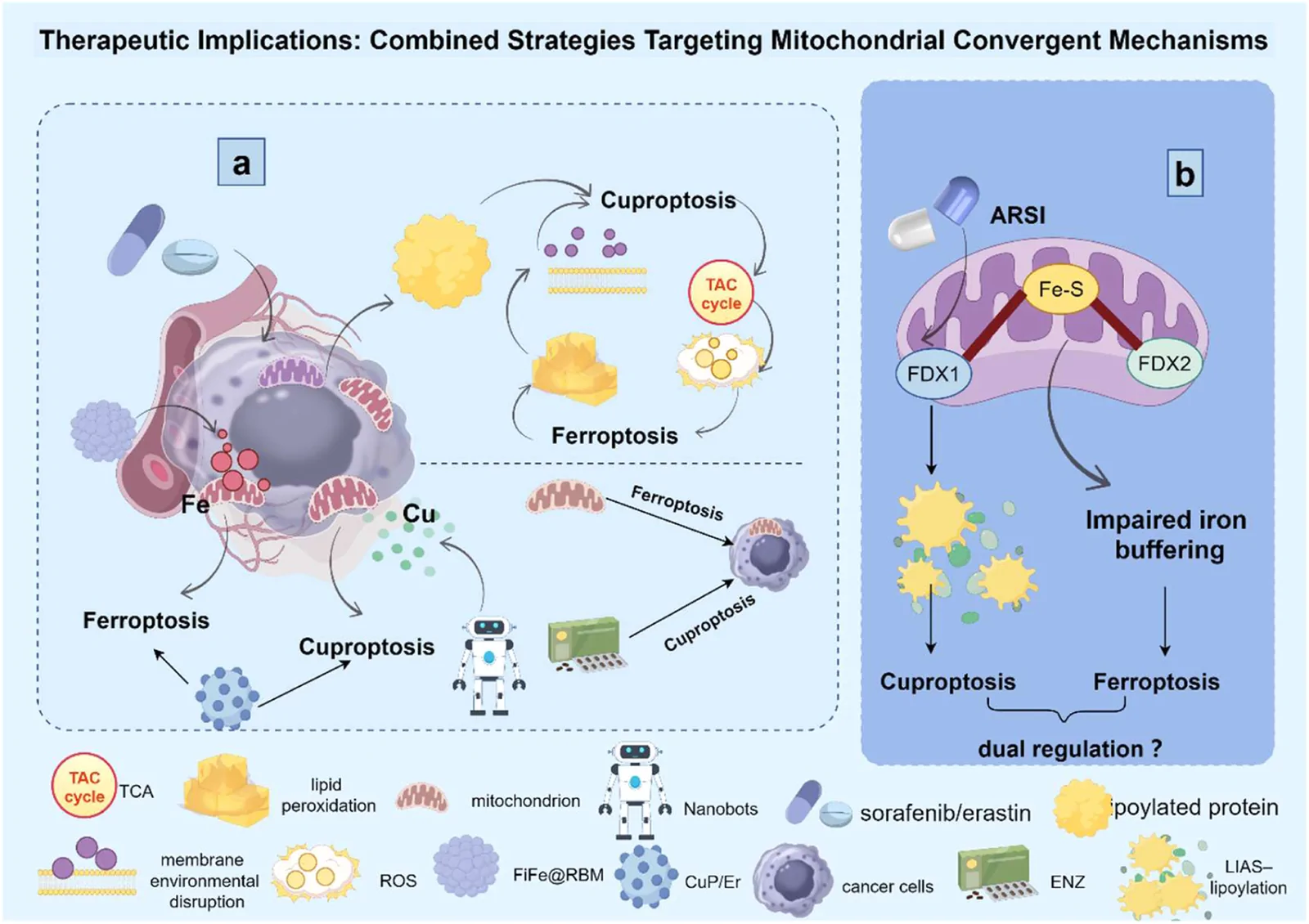
Fig. 4**a** Ferroptosis and cuproptosis may mutually reinforce each other at the mitochondrial level. Lipid peroxidation during ferroptosis can aggravate disruption of the membrane environment surrounding lipoylated enzymes targeted in cuproptosis, whereas cuproptosis-induced collapse of the TCA cycle may increase mitochondrial ROS production, thereby providing the oxidative drive required for ferroptosis. And the ferroptosis inducers sorafenib and erastin have been shown to enhance cuproptosis sensitivity by promoting copper-dependent aggregation of lipoylated proteins. Mitochondrial transplantation has also emerged as a potential strategy to increase sensitivity to erastin-induced ferroptosis.Notably, enzalutamide increases the reliance of CRPC cells on mitochondrial metabolism, thereby enhancing their susceptibility to ionophore-mediated cuproptosis. In parallel, nanotechnology-based approaches provide additional opportunities for therapeutic exploitation: FiFe@RBM biomimetic nanovesicles enable efficient targeting of CRPC cells for ferroptosis induction, Cu²⁺-loaded nanobots release copper in response to the acidic tumor microenvironment to trigger cuproptosis, and the core–shell nanoparticle CuP/Er co-delivers copper and erastin to coordinately activate cuproptosis and ferroptosis.Fig. 4**b** Therapeutic strategies targeting Fe–S cluster homeostasis can be conceptually divided into two translationally relevant directions. One strategy, exemplified by FDX2, aims to sensitize CRPC cells to ferroptosis by weakening Fe–S cluster assembly and impairing iron-buffering capacity. The other focuses on the FDX1–LIAS–lipoylation axis, exploiting the enhanced mitochondrial dependence induced by ARSIs to increase susceptibility to cuproptosis. By Figdraw.

The shared dependence on mitochondrial metabolic activity provides a common vulnerability background for both ferroptosis and cuproptosis, suggesting that simultaneous activation of these two death programs within the same CRPC cell may produce synergistic effects beyond the simple additive activity of single agents (Fig. 4). The theoretical basis for this strategy lies in the fact that, although ferroptosis and cuproptosis are fundamentally distinct at the molecular level, their damage signals may mutually amplify one another at the mitochondrial level. Lipid peroxidation during ferroptosis may aggravate disruption of the membrane environment surrounding lipoylated enzymes targeted in cuproptosis, whereas TCA cycle collapse induced by cuproptosis may increase mitochondrial ROS production, thereby providing the oxidative drive required for ferroptosis.

The strongest CRPC-specific evidence currently supports the concept that therapy-induced mitochondrial dependency can create vulnerability to metal-dependent cell death. Enzalutamide has been reported to increase the reliance of CRPC cells on mitochondrial metabolism, thereby enhancing their susceptibility to copper ionophore-mediated cuproptosis [41] (Fig. 4**a**). This finding provides direct CRPC evidence that ARSI-induced metabolic rewiring may expose a cuproptotic vulnerability. In parallel, ferroptosis-oriented strategies have also shown CRPC relevance. For example, biomimetic nanovesicles co-encapsulating a fatty acid synthase inhibitor and iron oxide nanoparticles (FiFe@RBM) efficiently target CRPC cells and release iron intracellularly, thereby inducing mitochondrial dysfunction, suppressing the AKT–mTOR pathway, and remodeling lipid metabolism toward PUFA-enriched phospholipid species that potentiate ferroptosis [82]. These studies support the feasibility of targeting ferroptosis or cuproptosis separately in CRPC. However, direct evidence for simultaneous ferroptosis–cuproptosis co-induction in CRPC remains lacking.

Several prostate cancer studies provide additional mechanistic support. Intercellular transfer of healthy mitochondria into aggressive prostate cancer cells such as PC-3 markedly enhances their sensitivity to erastin-induced ferroptosis, suggesting that enforced mitochondrial remodeling may provoke metabolic incompatibility or redox disequilibrium [83]. With respect to cuproptosis, Cu²⁺-loaded nanobots can release copper in response to the acidic tumor microenvironment. In vitro, these copper nanobots markedly promoted oligomerization of dihydrolipoamide S-acetyltransferase (DLAT), reduced the expression of proteins associated with Fe–S/lipoate machinery, including FDX1 and LIAS, and induced cuproptosis in human prostate cancer cells. In animal models, they achieved a tumor inhibition rate of approximately 75.75% [84]. These findings support the broader relevance of mitochondrial manipulation and copper-dependent death in prostate cancer, but their applicability to CRPC requires validation in therapy-resistant and patient-derived CRPC models.

Importantly, in hepatocellular carcinoma cells, ferroptosis inducers such as sorafenib and erastin enhance copper ionophore-induced cuproptosis by promoting copper-dependent aggregation of lipoylated proteins. Mechanistically, these agents increase protein lipoylation by inhibiting degradation of FDX1 mediated by a mitochondrial matrix-associated protease and simultaneously reduce GSH synthesis by suppressing cystine uptake, thereby weakening copper buffering and enhancing cuproptotic death [85]. In addition, CuP/Er core–shell nanoparticles co-deliver copper and erastin to coordinate cuproptosis and ferroptosis, induce immunogenic cell death, enhance antigen presentation, and improve the efficacy of immune checkpoint blockade in colorectal adenocarcinoma and triple-negative breast cancer models [86]. These studies demonstrate therapeutic co-induction and cross-tumor feasibility.

### 4.2 Nodal intervention centered on Fe–S cluster homeostasis: bidirectional regulation of FDX1/FDX2 and iron metabolism

Fe–S cluster homeostasis represents one of the most critical points of convergence between ferroptosis and cuproptosis. It not only sustains the activity of the mitochondrial electron transport chain, the TCA cycle, and multiple metabolic enzymes, but is also functionally coupled to the iron regulatory protein (IRP) system, thereby directly participating in the rebalancing of cellular iron uptake, storage, and utilization. Accordingly, disruption of Fe–S cluster biogenesis or maintenance does not affect a single enzymatic reaction in isolation; rather, it perturbs an integrated network encompassing mitochondrial metabolism, iron homeostasis, and redox buffering. This central positioning makes Fe–S cluster homeostasis an ideal intervention node linking ferroptosis and cuproptosis [70,87] (Fig. 4**b**) .Therapeutic strategies targeting Fe–S cluster homeostasis may be conceptually divided into two translationally relevant directions. The first, exemplified by FDX2, aims to sensitize CRPC cells to ferroptosis by weakening Fe–S cluster assembly and compromising iron homeostatic buffering capacity. The second centers on the FDX1–LIAS–lipoylation axis, leveraging the enhanced mitochondrial dependency induced by ARSIs to amplify susceptibility to cuproptosis [[88], [89]]. More intriguingly, these two strategies need not be considered mutually exclusive. In principle, if increased vulnerability to iron-dependent oxidative injury could be integrated in a temporally or dose-controlled manner with the preservation or exposure of the lipoylated target landscape required for cuproptosis, Fe–S cluster homeostasis might be transformed from a passive metabolic background into a therapeutic lever capable of simultaneously governing both ferroptosis and cuproptosis.At present, however, such bidirectional modulation in CRPC remains largely at the stage of mechanistic inference and preclinical conceptualization. In particular, direct combinatorial intervention studies targeting FDX1/FDX2 are still notably lacking, highlighting an important priority for future investigation.

## 5 Limitations and unresolved issues

Although the mitochondrial convergence model provides a useful framework for understanding ferroptosis and cuproptosis in CRPC, several important limitations and unresolved issues should be acknowledged. First, whether ferroptosis is intrinsically dependent on mitochondria remains context-dependent rather than universal. The canonical execution of ferroptosis is driven by iron-dependent lipid peroxidation of cellular membranes and is primarily controlled by lipid metabolism, iron availability, and antioxidant defense systems such as the SLC7A11–GSH–GPX4 axis. Mitochondria can shape ferroptosis sensitivity by regulating ROS production, TCA-cycle activity, electron transport, mitochondrial CoQ metabolism, iron handling, and lipid metabolic remodeling. However, mitochondria should not be interpreted as an obligatory execution organelle for all forms of ferroptosis. Therefore, in this review, mitochondrial involvement in ferroptosis is best understood as a threshold-modulating and context-shaping mechanism rather than as a universal requirement for ferroptotic cell death. Second, the current evidence base is uneven between ferroptosis and cuproptosis in CRPC. Compared with cuproptosis, ferroptosis has been more extensively studied in CRPC and therapy-resistant prostate cancer models. Several CRPC-specific or CRPC-related studies have implicated PHGDH inhibition, SLC7A11-mediated cystine uptake, FSP1, DECR1-dependent lipid metabolism, ACSL4-dependent lipid remodeling, and GPX4 compensation in ferroptosis sensitivity or ferroptosis resistance. Third, direct bidirectional crosstalk between ferroptosis and cuproptosis has not been firmly established in CRPC. The available evidence supports the existence of shared mitochondrial vulnerability nodes, including mitochondrial respiration, Fe–S cluster homeostasis, protein lipoylation, redox buffering, GSH metabolism, and mitochondrial quality control. However, direct evidence showing that cuproptosis-associated lipoylated protein aggregation or Fe–S cluster instability drives ferroptotic lipid peroxidation in the same CRPC model remains lacking. Conversely, whether ferroptotic membrane lipid peroxidation directly increases susceptibility to copper-induced mitochondrial proteotoxic stress in CRPC has not been experimentally demonstrated. Therefore, ferroptosis–cuproptosis interaction should currently be interpreted as a mechanistically plausible convergence model and therapeutic hypothesis rather than as a validated bidirectional amplification loop.Fourth, many mechanistic and therapeutic studies discussed in this review were derived from general prostate cancer models or non-prostate cancer models, including hepatocellular carcinoma, breast cancer, colorectal cancer, ovarian cancer, and other tumor systems. These studies provide valuable conceptual support, but they cannot directly establish CRPC-specific validity. In addition, commonly used prostate cancer cell lines and xenograft models may not fully capture the biological heterogeneity of CRPC, including AR-dependent CRPC, AR-indifferent or AR-plastic states, neuroendocrine differentiation, lineage plasticity, metabolic heterogeneity, and the influence of the tumor microenvironment. Future studies should therefore prioritize therapy-resistant CRPC cell lines, paired drug-sensitive and drug-resistant models, patient-derived organoids, xenografts, genetically engineered models, and clinical CRPC specimens. Fifth, the translational feasibility of targeting ferroptosis and cuproptosis in CRPC remains uncertain. Metal ions, copper ionophores, ferroptosis inducers, mitochondrial modulators, and nanodelivery systems may produce off-target toxicity, systemic metabolic disturbance, or inconsistent pharmacokinetic profiles. Moreover, reliable biomarkers are still needed to identify patients most likely to benefit from metal-dependent cell death-based therapies. Prospective validation using CRPC-specific preclinical models and, ultimately, clinical samples will be essential before these strategies can be translated into patient treatment.

Taken together, these limitations do not diminish the value of the mitochondrial convergence framework, but they define its current boundaries. At present, this framework should be viewed as a hypothesis-generating model that integrates mitochondrial metabolism, redox stress, metal homeostasis, and regulated cell death in CRPC. Future CRPC-focused studies are needed to determine whether these mitochondrial vulnerabilities can be converted into safe, selective, and clinically effective therapeutic strategies.

## 6 Conclusion and perspectives

Previous studies have often regarded mitochondria as a shared background or metabolic platform for ferroptosis and cuproptosis, implying that although both forms of metal-dependent cell death rely on mitochondrial metabolic activity, their mechanisms remain largely parallel and relatively independent. However, the present analysis suggests that mitochondria are far more than a passive backdrop; rather, they constitute a central hub at which ferroptosis and cuproptosis converge at the molecular, functional, and regulatory levels.Building on this convergence framework, we propose that CRPC may harbor a distinct mitochondrial-vulnerable therapeutic window. This window refers to a state in which, during the progression of castration-resistant prostate cancer and under the selective pressure of ARSIs, CRPC cells acquire a heightened dependence on mitochondrial function through metabolic reprogramming. On the one hand, these cells enhance oxidative phosphorylation, Fe–S cluster flux, and TCA cycle activity to sustain survival and proliferation. On the other hand, this very dependency places mitochondria in a precarious state of redox and proteostatic balance, thereby rendering CRPC cells significantly more susceptible to ferroptosis- and cuproptosis-inducing agents than normal cells or tumor cells that are less reliant on mitochondrial metabolism. Future studies should further define the quantitative thresholds that demarcate this therapeutic window, such as critical values for mitochondrial oxygen consumption rate, the GSH/GSSG ratio, and lipid peroxidation products. More importantly, prospective clinical studies will be required to determine whether therapeutic strategies guided by this mitochondrial-vulnerable window can translate into meaningful improvements in progression-free survival and overall survival for patients with CRPC.

## Institutional review board statement

Not applicable.

## Clinical trial registration number

Not applicable.

## Informed consent statement

Not applicable.

Supplementary materials.

Not available at present.

## Funding

This research was funded by the Institutional Research Project of the Guang'anmen Hospital, China Academy of Chinese Medical Sciences, grant number 2022s471; the Clinical Research and Achievement Transformation Capability Enhancement Project of High-Level Traditional Chinese Medicine Hospitals - Medical Institution Formulation R&D and New Drug Transformation Special Project, grant number HLCMHPP2023047; the Sixth National Program for Training Outstanding Clinical Talents in Traditional Chinese Medicine, grant number 8277823; and the Science and Technology Innovation Project of the China Academy of Chinese Medical Sciences, grant number CI2026A02212.

## CRediT authorship contribution statement

**Can-lin Wang:** Writing – original draft. **Jin-ting Lv:** Writing – review & editing, Writing – original draft. **Si-jia Liu:** Conceptualization, Data curation, Formal analysis, Funding acquisition, Writing – original draft, Writing – review & editing. **Yang-yang Zhang:** Writing – original draft, Writing – review & editing. **Shu-li Wang:** Conceptualization, Writing – original draft, Writing – review & editing. **Shu-qi Song:** Writing – review & editing, Writing – original draft.

## Declaration of competing interest

The authors declare that they have no known competing financial interests or personal relationships that could have appeared to influence the work reported in this paper.

## Data Availability

No new data were created or analyzed in this study.
